# New Insights on Hemp Oil Enriched in Cannabidiol: Decarboxylation, Antioxidant Properties and In Vitro Anticancer Effect

**DOI:** 10.3390/antiox10050738

**Published:** 2021-05-07

**Authors:** Anca Roxana Petrovici, Natalia Simionescu, Andreea Isabela Sandu, Vasile Paraschiv, Mihaela Silion, Mariana Pinteala

**Affiliations:** 1 Centre of Advanced Research in Bionanoconjugates and Biopolymers, “Petru Poni” Institute of Macromolecular Chemistry, 41A Grigore Ghica Voda Alley, 700487 Iasi, Romania; petrovici.anca@icmpp.ro (A.R.P.); natalia.simionescu@icmpp.ro (N.S.); sandu.isabela@icmpp.ro (A.I.S.); 2 SC OVVA IASI SRL, 707025 Baltati-Iasi, Romania; vasileparaschivro@gmail.com

**Keywords:** cannabidiol, hemp oil, soft decarboxylation, antioxidant properties, reactive oxygen species, apoptosis, osteosarcoma

## Abstract

This study aimed to obtain and characterize extracted hemp oil enriched in cannabidiol (CBD) by decarboxylation of cannabidiolic acid (CBDA) and to give new insights into its antioxidant and anticancer effects. Optimization of CBDA decarboxylation in hemp oil was performed, and CBD and CBDA contents and purities were determined by flash chromatography, ^1^H- and ^13^C-NMR. The antioxidant properties of CBD-enriched oil were investigated by Fe^2+^ chelating activity, Fe^3+^ reducing antioxidant power assay, O_2_^●−^ scavenging activity, HO^●^ scavenging ability and lipid peroxidation inhibitory assay, and its cytotoxicity, apoptosis- and oxidative stress-inducing effects on NHDF, MeWo, HeLa, HepG2 and HOS cells were determined. The CBD concentration in hemp oil was increased by CBDA soft decarboxylation optimized at 90 °C, for 1 h and the resulting oil was capable of reducing iron, scavenging free radicals and inhibiting lipid peroxidation in cell-free oxidative conditions. CBD-enriched oil promoted NHDF proliferation at up to 15 µg CBD/mL, while inducing apoptosis and ROS production and modulating antioxidant enzymes’ gene expression in cancer cells, being selective for osteosarcoma cells, and induced apoptosis by p53- and ROS-independent mechanisms. CBD-enriched hemp oil demonstrated antioxidant properties in oxidative conditions and promoted normal fibroblasts’ proliferation, while inducing apoptosis and ROS production in cancer cells.

## 1. Introduction

*Cannabis sativa* L. or hemp was one of the first plants used to obtain fibers, oil and biomass and is cultivated specifically for its industrial uses. In addition, hemp-derived products are used as medicine, cosmetics, biofuel and animal feed, as well as for paper, varnishes, inks and phytoremediation [1]. The production of *C. sativa* L. and its derived products is regulated by European legislation, which states that hemp cultivation is permitted only if it contains less than 0.2% (*w/w*) Δ9-tetrahydrocannabinol (THC) due to its psychoactive effects (Regulation 1307/2013). Like in most European countries, hemp products are legal in Romania as long as their THC concentration is below 0.2%. In addition to THC, there are more than 100 other active compounds in hemp [2,3,4], and it was observed from in vitro and in vivo studies that hemp extracts exhibit significant synergistic biological effects, as compared to their individual compounds, inhibiting cancer cells’ proliferation, angiogenesis and metastasis, as well as promoting apoptosis [5]. However, non-cancerous cells respond differently to cannabinoids, their viability remaining unchanged or even being increased [6].

On the other hand, the main targets of cancer treatments are the reduction in tumor sizes, the inhibition of angiogenesis and metastasis and, primarily, the induction of controlled cancer cell death, i.e., apoptosis. The principal issue of chemotherapeutic agents, however, is that they commonly have severe side effects, attacking normal, healthy cells as well as cancer cells. This issue could be mitigated by the use of natural compounds or mixtures, which have very few and mild side effects while exhibiting anticancer properties and/or alleviating chemotherapy fallouts.

The main proposed mechanism of action for cannabinoids in cancer cells is the induction of apoptosis by stimulating the production of reactive oxygen species (ROS) [6]. It is well known that ROS, such as superoxide anion radical (O_2_^●−^), hydroxyl radical (HO^●^) or hydrogen peroxide (H_2_O_2_), are produced in living cells as secondary metabolic products and are required for normal cell function at physiological concentrations [7]. H_2_O_2_ activates various redox signaling pathways and exhibits either pro- or anti-apoptotic activities, depending on its concentration and cellular localization [8].

It should be pointed out that also several pre-clinical in vitro and in vivo studies showed that among discovered cannabinoids, cannabidiol (CBD) alone or combined with standard chemotherapeutic drugs reduces cancer cells’ viability and tumor size, making it the most promising cannabinoid used to treat various diseases, including cancer [1]. Moreover, CBD is a non-toxic and non-psychoactive cannabinoid that has a wide array of pharmacological effects, including antioxidant and anti-inflammatory, as well as analgesic, antiemetic and appetite-stimulating effects [9]. Very important to emphasize, CBD is known to have a very low affinity for CB1 and CB2 receptors of the endocannabinoid system, being non-psychoactive, but its exact mechanism of action is still unclear [6].

Based on existing information, it can be said that hemp oil enriched in CBD could become one of the most powerful nutraceuticals recommended as adjuvants in numerous diseases, as well as for treatment for pain and in the reduction of chemotherapy side effects [1]. In addition, hemp extracts containing cannabinoids (including CBD) were classified as novel foods in the European Union (Regulation 2015/2283 on Novel Food). Hemp oils of different CBD concentrations with numerous health benefits and very few side effects are commercially available, and are easy to characterize and obtain by different extraction methods. Moreover, the concentration of CBD in hemp oil can be increased by the decarboxylation of the main cannabinoid found in hemp, cannabidiolic acid (CBDA). CBDA is transformed to CBD through a slow decarboxylation process which takes place spontaneously at room temperature in plants, and is accelerated by extended storage, light exposure and higher temperatures [10].

This study aimed to obtain and characterize hemp oil enriched in CBD for use as a nutraceutical in cancer management and treatment. For this purpose, we determined the most efficient decarboxylation conditions of CBDA from extracted hemp oil. Furthermore, we investigated the cell-free antioxidant properties of CBD-enriched hemp oil (ferrous ions’ chelating activity, ferric ions reducing antioxidant power assay, O_2_^●−^ scavenging activity, HO^●^ scavenging ability and lipid peroxidation inhibitory assay), as well as its effects on normal dermal fibroblasts (NHDF), malignant melanoma (MeWo), adenocarcinoma (HeLa), hepatocellular carcinoma (HepG2) and osteosarcoma (HOS) cells, with emphasis on cytotoxicity, induction of apoptosis and oxidative stress. The results show that hemp oil enriched in CBD exhibits antioxidant properties in oxidative conditions and has significant anticancer effects on MeWo, HeLa, HepG2 and HOS cells, while being safe for normal fibroblasts, thus suggesting its potential application as an adjuvant in cancer therapy.

## 2. Materials and Methods

### 2.1. C. sativa L. Plant Growth

All extracts were done from a single strain of *C. sativa* L. denoted KC Dora. Plants were cultivated on an agricultural field, in north-eastern Romania, following EU and country legislation (EU Regulation 1307/2013, Romanian Law 339/2005). They were harvested when 50% of them were flowering and then air-dried in the dark.

### 2.2. Hemp Oil Extraction

For this study, 500 g of the top third of the plant was used. The extraction procedure involved maceration of the finely ground plant in ethanol (ratio between dried plants and ethanol: 1:5) at room temperature for 30 min, under slow stirring. After filtration, the solid residue was washed again with ethanol at a ratio of 1:2 for 10 min and then filtered. The two supernatant solutions were combined and evaporated under vacuum, at 150 mbar, 55 °C, until the ethanol had been removed. The resulting hemp oil was kept at 4 °C until use.

### 2.3. CBD Purification and Characterization

The hemp oil was processed by flash chromatography using a column of appropriate diameter and packed 1/3 with appropriate silica gel (230–400 mesh). A sample of 60 mg crude hemp oil was diluted with methanol and slowly introduced on the silica bed using the column wall as support. The elution of other compounds was performed with ethanol:acetonitrile 9:1, followed by methanol only for CBD elution.

The final fraction was collected and freeze-dried, obtaining 5 mg of solid white compound, representing purified CBD, from 60 mg crude hemp oil. The purity of the compound was verified by ^1^H- and ^13^C-NMR, using a Bruker Avance NEO 400 MHz Spectrometer, equipped with a 5 mm inverse detection z-gradient probe, operating at 400.1 and 100.6 MHz for ^1^H and ^13^C nuclei. The sample was dissolved in dimethyl sulfoxide-d_6_ (DMSO-d_6_) and the spectra were recorded at room temperature. Chemical shifts were reported in ppm and referred to residual solvent signal (ref. ^1^H 2.512 ppm and ^13^C 39.47 ppm).

**Figure 1:**
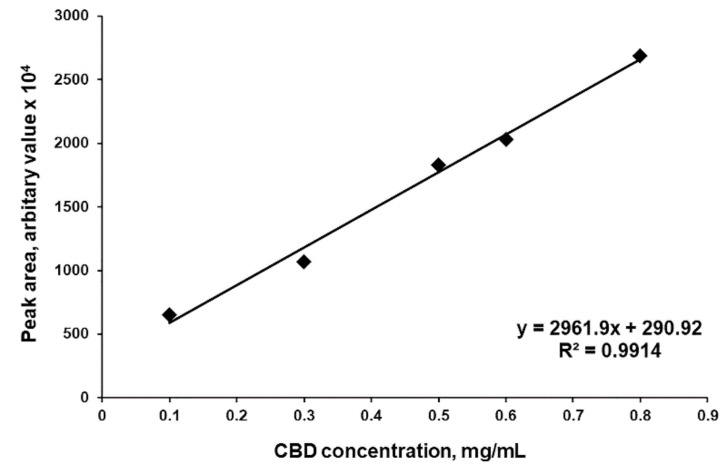
Representative CBD calibration curve.

The HPLC determination of CBDA and CBD from crude and decarboxylated hemp oil samples was performed using a Perkin Elmer HPLC system with a Flexar UV/VIS Detector at 220 nm and 30 °C. A 10 µL fixed injection loop was made in a Mediterranea Sea 18 column (5 µm, 250 mm × 4.6 mm) with a 1 mL/min flow mobile phase in a gradient formed by A: Milli-Q H_2_O with 0.1% HCOOH, pH 2, and B: methanol [11] with 0.1% HCOOH. The optimum conditions for analysis were as follows: phase B increased linearly over 11 min from 50 to 80% and then to 95% over the next 2 min, maintained for 3 min, then returned at 50% in 2 min, followed by 12 min of equilibration before the next injection [12]. In order to obtain the calibration curve for CBD, a stock solution of 1 mg/mL pure CBD in methanol was prepared and serial dilutions were made (0.1, 0.3, 0.5, 0.6 and 0.8 mg/mL). The solutions were analyzed by HPLC and the CBD concentration was plotted against peak area (Figure 1).

By comparing two HPLC spectra of the same concentration of CBD and CBDA, respectively, the same peak areas were obtained. Due to this observation, CBDA concentrations were calculated using the same equation from the CBD calibration curve.

### 2.4. Decarboxylation of CBDA from Hemp Oil

In this study, the decarboxylation conditions were varied to screen for the best conditions to obtain maximum CBDA decarboxylation yield. Therefore, hemp oil was incubated at 500 mbar, 60 rpm, varying the temperature and time: (1) 70 °C, 1 h; (2) 70 °C, 2 h; (3) 70 °C, 3 h; (4) 70 °C, 4 h; (5) 80 °C, 1 h; (6) 80 °C, 2 h; (7) 80 °C, 3 h; (8) 80 °C, 4 h; (9) 90 °C, 1 h; (10) 90 °C, 2 h; and (11) 100 °C, 1 h.

After decarboxylation, each sample was analyzed by HPLC using the method described in Section 2.3, peak areas were determined and concentrations of CBD and CBDA were calculated using the equation of the calibration curve (Section 2.3). Decarboxylated hemp oil with maximum CBDA transformation efficiency was considered as CBD-enriched hemp oil, standardized as μg CBD/mL and used in the following experiments.

### 2.5. In Vitro Antioxidant Activity Evaluation

#### 2.5.1. Ferrous Ions’ (Fe^2+^) Chelating Activity

The chelating activities of CBD-enriched hemp oil, pure CBD and crude hemp oil compared to gallic acid (as a common antioxidant reference) were estimated using a previously described method [13], with some modifications. In brief, CBD-enriched hemp oil (15 μg CBD/mL), pure CBD, crude hemp oil and gallic acid were dissolved in 200 μL methanol and 100 μL ferrous chloride tetrahydrate (FeCl_2_ × 4 H_2_O, 2 mM) was added. Methanol was used as a control. Then, 200 μL ferrozine (2 mM) was added and the total volume was adjusted to 2 mL with ethanol. The mixture was vigorously shaken and incubated at room temperature for 10 min. The Fe^2+^ chelating activity was estimated by measuring the absorbance of the Fe^2+^-ferrozine complex at 562 nm in a 96-well plate using a FLUOstar^®^ Omega microplate reader (BMG LABTECH, Ortenberg, Germany). The inhibition percentage of Fe^2+^–ferrozine complex was calculated using the following formula: ${\mathrm{Fe}}^{2+}\;\mathrm{chelating}\;\mathrm{effect}\;\left(\%\right)=\left(1-\frac{\mathrm{As}}{\mathrm{Ac}}\right)\times 100$, where Ac is the control’s absorbance and As is the sample absorbance [13].

#### 2.5.2. Ferric Ions (Fe^3+^) Reducing Antioxidant Power (FRAP) Assay

FRAP assay was performed using a protocol adapted from Li et al. [14] for CBD-enriched hemp oil, pure CBD and crude hemp oil compared to gallic acid (as a common antioxidant reference). Different concentrations of CBD-enriched hemp oil (15–45 μg CBD/mL), pure CBD, crude hemp oil and gallic acid were dissolved in 50 μL methanol, mixed with 650 μL sodium phosphate buffer (Na_2_HPO_4_/KH_2_PO_4_, 0.2 M, pH 6.6) and 650 μL potassium ferricyanide (K_3_Fe(CN)_6_, 1%) and the mixture was incubated for 20 min at 50 °C. After 20 min, 650 μL trichloroacetic acid (10%) was added to the mixture and 910 μL of this solution was mixed with 910 μL distilled water and 180 μL ferric chloride (FeCl_3_, 0.1%). The absorbance was measured at 700 nm in a 96-well plate using a FLUOstar^®^ Omega microplate reader (BMG LABTECH, Ortenberg, Germany). The reducing power (%) was expressed as the ratio between the absorbance for 15 μg CBD/mL and the absorbance for 45 μg CBD/mL.

#### 2.5.3. Superoxide Anion Radical (O_2_^●−^) Scavenging Activity

The abilities of CBD-enriched hemp oil, pure CBD and crude hemp oil compared to gallic acid (as a common antioxidant reference) to scavenge O_2_^●−^ were determined with a method adapted after Xiong et al. [15]. In brief, CBD-enriched hemp oil (15 μg CBD/mL), pure CBD, crude hemp oil and gallic acid were dissolved in 40 μL methanol, mixed with 1.8 mL Tris-HCl (0.05 M, pH 8) and incubated for 20 min at 25 °C on a thermo-shaker. Methanol was used as a control. Next, 160 μL of pyrogallol (25 mM) was added and the mixture was incubated at 25 °C on a thermo-shaker for 5 min. Then, 10 μL HCl (8 M) was added to complete the reaction and the absorbance was measured at 325 nm in a 96-well plate using a FLUOstar^®^ Omega microplate reader (BMG LABTECH, Ortenberg, Germany). The scavenging percentage was calculated using the formula: $\mathrm{Scavenging}\;\mathrm{rate}\;\left(\%\right)=\left(\frac{\mathrm{Ac}\;-\mathrm{As}}{\mathrm{As}}\right)\times 100$, where Ac is the control’s absorbance and As is the sample’s absorbance [15].

#### 2.5.4. Hydroxyl Radical (HO^●^) Scavenging Ability

HO^●^ scavenging abilities of CBD-enriched hemp oil, pure CBD and crude hemp oil compared to gallic acid (as a common antioxidant reference) were determined using a previously published method [15], with some modifications, as follows: CBD-enriched hemp oil (15 μg CBD/mL), pure CBD, crude hemp oil and gallic acid were dissolved in 400 μL methanol and mixed vigorously with 400 μL O-phenanthroline (2.5 mM) and 400 μL phosphate-buffered saline (PBS, 0.2 M, pH 7.4). Then, 400 μL of ferrous sulfate heptahydrate (FeSO_4_ × 7 H_2_O, 2.5 mM) and 400 μL of H_2_O_2_ were added and the mixture was incubated at 37 °C on a thermo-shaker for 1 h. After incubation, the absorbance was measured at 536 nm in a 96-well plate using a FLUOstar^®^ Omega microplate reader (BMG LABTECH, Ortenberg, Germany). Methanol was used as a control. The scavenging percentage of HO^●^ was calculated with the formula: $\mathrm{Scavenging}\;\mathrm{rate}\;\left(\%\right)=\frac{\mathrm{As}\;-A1}{A0-A1}\times 100$, where As is the absorbance of the sample; A0 is the absorbance of distilled water in reaction and A1 is the absorbance of H_2_O_2_ in reaction [15].

#### 2.5.5. Lipid Peroxidation Inhibitory Assay

Lipid peroxidation inhibitory activities of CBD-enriched hemp oil, pure CBD and crude hemp oil compared to gallic acid (as common antioxidant reference) were measured by a previously described method [16], with some modifications. CBD-enriched hemp oil (15 μg CBD/mL), pure CBD, crude hemp oil and gallic acid were dissolved in 100 μL methanol and mixed with 900 μL phosphate buffer (dipotassium hydrogen phosphate in distilled water 0.2 M at pH 7) and 1000 μL linoleic acid emulsion (which was prepared by mixing 155 μL linoleic acid and 175 μg Tween-20 in 50 mL phosphate buffer 0.2 M). Methanol was used as a control. The mixture was incubated at 37 °C and 50 μL of this solution was taken after 25 min, and every 24 h for three days, and mixed with 1.85 mL ethanol and 50 μL FeCl_2_ × 4 H_2_O solution (20 mM in 3.5% HCl). The resulting solution was mixed thoroughly and 50 μL potassium thiocyanate (KSCN) solution (30% in distilled water) was added. The absorbance of the resulting clear solution (50 μL) was recorded at 500 nm in a 96-well plate using a FLUOstar^®^ Omega microplate reader (BMG LABTECH, Ortenberg, Germany). The inhibitory effect was calculated using the formula: $\mathrm{Inhibition}\;\mathrm{effect}\;\left(\%\right)=\left(\frac{\mathrm{As}-\mathrm{Ac}}{\mathrm{As}}\right)\times 100$, where As is the absorbance of the sample and Ac is the control’s absorbance [16].

### 2.6. Cell Culture

Normal dermal fibroblasts (NHDF, purchased from PromoCell, Heidelberg, Germany), malignant melanoma (MeWo), adenocarcinoma (HeLa), hepatocellular carcinoma (HepG2) and osteosarcoma (HOS) cells (all malignant cell lines purchased from CLS Cell Lines Service GmbH, Eppelheim, Germany) were grown in alpha-MEM medium (Lonza, Basel, Switzerland) supplemented with 10% fetal bovine serum (FBS, Gibco, Thermo Fisher Scientific, Waltham, MA USA) and 1% Penicillin-Streptomycin-Amphotericin B mixture (10 K/10 K/25 μg, Lonza, Basel, Switzerland).

### 2.7. Cytotoxicity Assay (MTS)

Cytotoxicity was measured using the CellTiter 96^®^ AQueous One Solution Cell Proliferation Assay (Promega, Madison, WI USA), according to the manufacturer instructions. Cells were seeded at a density of 0.5 × 10^5^ (NHDF) or 1 × 10^5^ cells/mL (malignant cell lines) into 96-well tissue culture-treated plates. After 24 h the medium in each well was replaced with 100 μL fresh complete medium (control, 0 µg CBD/mL) or various concentrations of CBD-enriched hemp oil (5, 10, 15, 20, 25 and 30 µg CBD/mL). CBD-enriched hemp oil samples used for cell treatments were obtained by serial dilution of hemp oil with maximum yield of CBDA decarboxylation. Cells were treated for 48 h and 20 µL MTS reagent was added 1–3 h prior to absorbance readings at 490 nm on a FLUOstar^®^ Omega microplate reader (BMG LABTECH, Ortenberg, Germany). Experiments were done in triplicate and treated cells’ viability was expressed as a percentage of control cells’ viability. The CBD-enriched hemp oil concentration that induced a 50% decrease in cell viability (IC_50_) was determined with GraphPad Prism 8 software from the dose–response curves for each cell line. The selectivity index of CBD-enriched hemp oil was calculated using the formula: SI = IC_50_ normal cells/IC_50_ cancer cells, as suggested by Badisa et al. [17].

### 2.8. Morphological Analysis

Cells were seeded at a density of 0.5 × 10^5^ (NHDF) or 1 × 10^5^ cells/mL (MeWo, HeLa, HepG2 and HOS) into 24-well tissue culture-treated plates, allowed to adhere overnight, then incubated with fresh complete medium (control, 0 µg CBD/mL) or various concentrations of CBD-enriched hemp oil (5, 10, 15, 20 and 25 µg CBD/mL) for 48 h. CBD-enriched hemp oil samples used for cell treatments were obtained by serial dilution of hemp oil with maximum yield of CBDA decarboxylation. After incubation, the cells were visualized in bright field using a DMI 3000 B inverted microscope (Leica, Wetzlar, Germany). Experiments were done in triplicate and images were acquired using the 10× objective for assessing morphological changes.

### 2.9. Acridine Orange/Ethidium Bromide (AO/EB) Staining for Apoptosis

A modified method of Ribble et al. [18] for AO/EB staining of cells was applied. Cells were seeded at a density of 0.5 × 10^5^ (NHDF) or 1 × 10^5^ cells/mL (MeWo, HeLa, HepG2 and HOS) into 24-well tissue culture-treated plates, allowed to adhere overnight, then incubated with fresh complete medium (control, 0 µg CBD/mL) or various concentrations of CBD-enriched hemp oil (5, 10, 15, 20 and 25 µg CBD/mL) for 48 h. CBD-enriched hemp oil samples used for cell treatments were obtained by serial dilution of hemp oil with maximum yield of CBDA decarboxylation. After incubation, the medium was removed and cells were stained with AO/EB solution (4 µg AO/EB/mL) for 1 min, then washed with PBS and visualized in complete medium, using a DMI 3000 B inverted microscope (Leica, Wetzlar, Germany). Experiments were done in triplicate and images were acquired using the I3 filter and the 20× objective.

### 2.10. Evaluation of Intracellular H_2_O_2_ Production

Intracellular H_2_O_2_ production was measured using 2′,7′-dichlorodihydrofluorescein diacetate (DCFH-DA), as previously described [19]. Cells were seeded at a density of 0.5 × 10^5^ (NHDF) or 1 × 10^5^ cells/mL (MeWo, HeLa, HepG2 and HOS) into 12-well tissue culture-treated plates, allowed to adhere overnight, then incubated for 24 h with fresh complete medium (control) or specific concentrations of CBD-enriched hemp oil (IC_50_ calculated for each cell line). After incubation, cells were loaded with 2 µM DCFH-DA for 20 min at 37 °C. After a gentle wash, the cells were scraped in a serum-free medium and the fluorescence was measured at 485/520 nm in 96-well black plates using a FLUOstar^®^ Omega microplate reader (BMG LABTECH, Ortenberg, Germany). Experiments were done in triplicate, and H_2_O_2_ production was calculated as relative fluorescence units (RFU)/mg total cellular protein and expressed as fold change of control values. Total cellular protein was determined using the bicinchoninic acid assay.

### 2.11. RNA Isolation and Gene Expression Analysis

Cells were seeded at a density of 0.625 × 10^5^ (NHDF) or 1.25 × 10^5^ cells/mL (MeWo, HeLa, HepG2 and HOS) into 6-well tissue culture-treated plates, allowed to adhere overnight, then incubated with fresh complete medium (control) or specific concentrations of CBD-enriched hemp oil (IC_50_ calculated for each cell line) for 48h. After incubation, cells were rinsed once with PBS and total RNA was isolated using TRIzol reagent (Invitrogen, Thermo Fisher Scientific, Waltham, MA USA), according to the manufacturer’s protocol. Reverse-transcription (RT) of RNA was performed on a Veriti PCR system, using the High Capacity cDNA Reverse Transcription kit and random RT primers (all from Applied Biosystems, Thermo Fisher Scientific, Waltham, MA USA). Real-time quantitative PCR reactions were performed on a QuantStudio 12K Flex Real-Time PCR System (Applied Biosystems, Thermo Fisher Scientific, Waltham, MA USA), using custom primers (Table 1) for B-cell lymphoma protein 2 (BCL2)-associated X (BAX), BCL2 apoptosis regulator (BCL2), tumor protein p53 (TP53), MDM2 proto-oncogene (MDM2), superoxide dismutase 1 (SOD1), catalase (CAT), glutathione peroxidase 1 (GPX1), glutathione-disulfide reductase (GSR) and 18S as a reference gene (Invitrogen, Thermo Fisher Scientific, Waltham, MA USA) and the SyBr Select Real-Time PCR Master Mix (Applied Biosystems, Thermo Fisher Scientific), according to the manufacturer’s instructions. Experiments were done in triplicate and, for each sample, duplicate measurements were done in 96-well reaction plates. Obtained data were analyzed using the QuantStudio 12K Flex Software v1.2 (Applied Biosystems, Thermo Fisher Scientific, Waltham, MA USA) with the automatic Cq setting. The expression level of each gene of interest was determined relative to 18S (as a reference gene) and calculated using the 2^−ΔΔCq^ method [20].

### 2.12. Statistical Analysis

Statistical analysis was performed using GraphPad Prism 8 software (GraphPad Software Inc., San Diego, CA, USA). Data were expressed as means ± standard error of the mean and analyzed by independent two-tailed (Student’s) *t*-test or one-way ANOVA with Geisser­–Greenhouse correction and Dunnett’s multiple comparisons test, considering *p* < 0.05 statistically significant.

## 3. Results and Discussion

### 3.1. Hemp Oil Extraction, Purification and Characterization

Many published methods for oil extraction from hemp with different efficiencies for cannabinoids’ enrichment are used. Ethanol extraction without degradation of biologically active compounds at room temperature seems to be the most efficient method of recovering cannabinoids from hemp [21]. Therefore, oil extraction with ethanol at room temperature from *C. sativa* L. denoted KC Dora, cultivated in north-eastern Romania (see Section 2.1), was performed, followed by the evaporation of the solvent under vacuum, at 55 °C, resulting in an extraction yield of 5.09% (from 1 g vegetal material was obtained 50.9 mg hemp oil).

**Figure 2:**
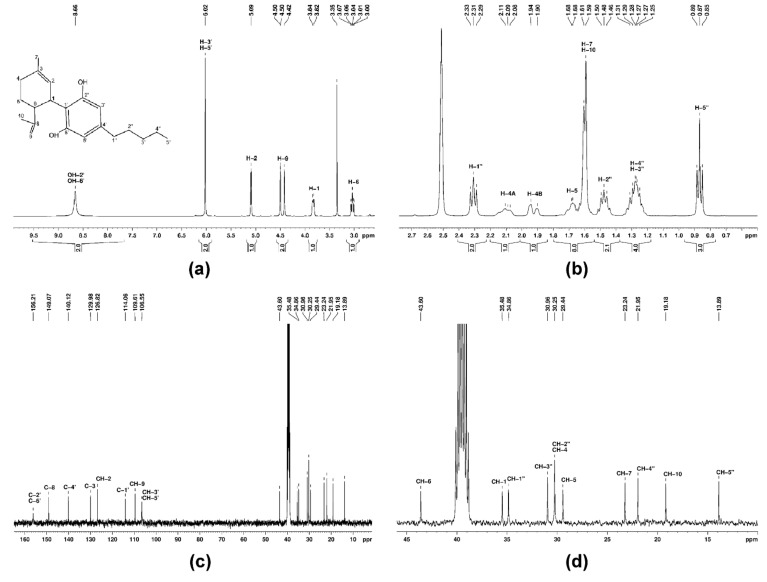
NMR spectra of pure CBD in DMSO-d_6_: (**a**) ^1^H-NMR spectra in the range of δ 10–2.5 ppm; (**b**) ^1^H-NMR spectra in the range of δ 2.8–0.5 ppm; (**c**) full ^13^C-NMR spectra; (**d**) ^13^C-NMR spectra in the range of δ 45–10 ppm.

A sample of 60 mg crude hemp oil was processed by flash chromatography (see Section 2.3) in order to separate the component compounds of the oil. The last collected eluent fraction was freeze-dried, obtaining 5 mg of solid white compound, representing purified CBD. The purity of the compound was verified by ^1^H- and ^13^C-NMR in DMSO-d_6_ solvent (Figure 2). The NMR assignments were done according to literature data [22]: ^1^H-NMR (400 MHz, DMSO-d_6_, δ (ppm)): 0.87 (3H, t, *J* = 8.0 Hz, H-5″), 1.25–1.31 (4H, m, H-3″ and H-4″), 1.48 (2H, q, *J* = 8.0 Hz, H-2″), 1.59–1.68 (8H, m, CH_3_-7, CH_3_-10 and H-5), 1.90–2.11 (2H, m, H-4), 2.31 (2H, t, *J* = 8.0 Hz, H-1″), 3.04 (1H, t, *J* = 8.0 Hz, H-1″), 3.83 (1H, d, *J* = 8.0 Hz, H-1), 4.42–4.45 (2H, m, H-9), 5.09 (1H, s, H-2), 6.02 (2H, s, H-3′, H-5′) and 8.66 (2H, s, OH-2′, OH-6′) and ^13^C-NMR (100 MHz, DMSO-d_6_, δ (ppm)): 13.9 (CH-5″), 19.2 (CH_3_-10), 21.9 (CH-4″), 23.2 (CH_3_-7), 29.4 (CH-5), 30.2 (CH-4 and CH-2″), 30.9 (CH-3″), 34.9 (CH-1″), 35.5 (CH-1), 43.6 (CH-6), 106.6 (CH-3′ and CH-5′), 109.6 (CH-9), 114.1 (C-1′), 126.8 (CH-2), 129.9 (C-3), 140.1 (C-4′), 149.1 (C-8) and 156.2 (C-2′ and C-6′).

**Figure 3:**
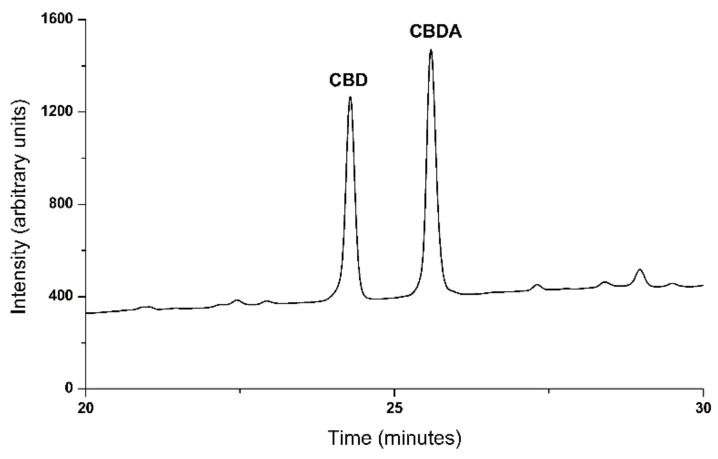
Representative HPLC chromatogram for crude extracted hemp oil. CBD: cannabidiol, CBDA: cannabidiolic acid.

HPLC analysis of the extracted crude hemp oil revealed 9.14% (*w/w*) CBD and 13.54% (*w/w*) CBDA (Figure 3).

### 3.2. Decarboxylation of CBDA from Hemp Oil

As it was mentioned before, CBDA is the most abundant cannabinoid in *C. sativa* L. plants, and is produced by cannabidiolic acid synthase from cannabigerolic acid. Through a slow decarboxylation process, which takes place spontaneously at room temperature in plants, CBDA is partially transformed into CBD [10]. Moreover, once the ambient temperature was increased, a more effective decarboxylation process was observed, yielding a higher content of CBD, a neutral cannabinoid which is the pharmacologically active compound in hemp [10].

**Figure 4:**
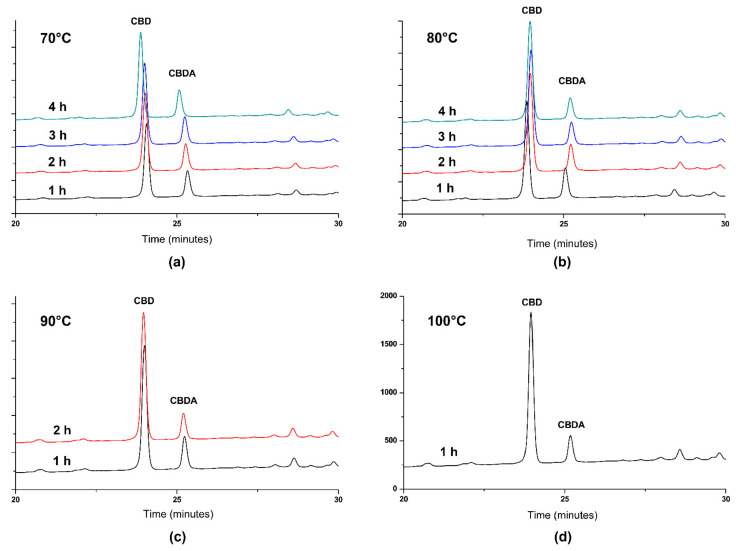
Representative HPLC chromatograms for decarboxylated hemp oil in different soft conditions: (**a**) 70 °C; (**b**) 80 °C; (**c**) 90 °C; (**d**) 100 °C. CBD: cannabidiol, CBDA: cannabidiolic acid.

In order to obtain a therapeutically valuable product, characterized by a higher concentration of CBD compared to CBDA, CBDA in hemp oil could be subjected to a decarboxylation reaction. For this purpose, Citti et al. [10] performed the decarboxylation of CBDA from hemp seed oil and compared the CBD yield after decarboxylation of CBDA in an open reactor at 80–120 °C and in a closed reactor at 120 °C. This study revealed that if the decarboxylation process takes place at high temperatures (over 100 °C), a significant loss (up to 60%) of the total concentration of CBDA and CBD is observed, denoting that it is an aggressive process on the extract and could lead to the degradation of bioactive molecules [10]. In order to determine the maximum transformation yield of CBDA into CBD without degradation, in the present study, the decarboxylation reactions were done in different soft conditions (70–100 °C, 1–4 h, 500 mbar, 60 rpm) (see Section 2.4). The resulting hemp oil samples were analyzed by HPLC (Figure 4), and the concentrations of CBD and CBDA were calculated using the same equation of the calibration curve (Figure 1), due to the fact that the molar extinction coefficient of both compounds at 220 nm is the same. Decarboxylated hemp oil with maximum CBDA transformation efficiency was considered as CBD-enriched hemp oil, standardized as μg CBD/mL and used in the following experiments. The decarboxylation conditions and their respective results are summarized in Table 2. Data have shown that the highest content of CBD in hemp oil, together with the highest yield of CBDA decarboxylation, is obtained after treating the hemp oil at 90 °C at 60 rpm and 500 mbar. Moreover, reaction time did not affect CBD and CBDA yield and concentration significantly: after 1 and 2 h of decarboxylation at 90 °C at 60 rpm and 500 mbar, yields of 95.72 and 95.28%, respectively, were obtained from initial concentrations. The transformation from CBDA to CBD was 66.62% for 1 h reaction time and 73.41% for 2 h reaction time, obtaining 17.2% (*w/w*) CBD and 4.52% (*w/w*) CBDA after 1 h and 17.82% (*w/w*) CBD and 3.8% (*w/w*) CBDA after 2 h, respectively, representing 8.6 and 8.91 mg CBD/g vegetal material, which is 35.4 and 36.5 times higher than reported for hemp seed oil (0.244 mg CBD/g vegetal material) [23]. Decarboxylation reactions performed at temperatures lower than 90 °C were less effective (around 68% CBDA transformation yield), while at 100 °C and 1 h reaction time, the transformation yield was 80.06%, but the CBD concentration slightly decreased due to degradation processes, specific for higher temperatures as demonstrated by Citti et al. [10].

### 3.3. In Vitro Antioxidant Activity of CBD-Enriched Hemp Oil

In the literature, CDB’s antioxidant role is based on two main mechanisms which are specific for phenol derivatives: one is based on electron transfer to reduce any compound and the second one is hydrogen donation to quench free radicals [9,24]. These two mechanisms were confirmed also by quantum chemical approaches [24], and they pointed out that CBD has a good antioxidant profile and its chemical structure can inhibit oxidative stress by free radical scavenging. Besides, it should be mentioned that in order to prevent the chain radical reactions, the newly formed radicals have to be stable and, to this end, Borges and da Silva [24] have shown that the stabilization of formed radicals of CBD is higher than that of nonphenolic compounds.

In brief, to determine the antioxidant properties of natural extracts, in the literature there are many methods based on the two mentioned main mechanisms: one is the reduction of a metallic species (e.g., ferric ions) by the natural compound, and another is the reaction of the natural compound with a free radical, such as O_2_^●−^ or HO^●^ [25]. In order to properly evaluate the antioxidant capacity of a natural extract or compound, both mechanisms should be investigated and at least two methods should be applied.

In our present work, the antioxidant activity of CBD-enriched hemp oil, pure CBD, crude hemp oil and gallic acid was determined by using different methods addressing the two mentioned mechanisms: Fe^2+^ chelating activity, FRAP assay, O_2_^●−^ scavenging activity, HO^●^ scavenging ability and lipid peroxidation inhibitory assay (see Section 2.5), and the results are summarized in Table 3.

The ability of natural compounds to chelate iron represents a valuable antioxidant property by hindering metal-catalyzed oxidation. In this context, Gülçin et al. [13] demonstrated that Fe^2+^ cations are powerful oxidants, involved in lipid oxidation processes, and generate ROS *in vivo*; therefore, Fe^2+^ chelators could offer protection against oxidative damage, while Fe^3+^ cations are predominant in foods and produce radicals from peroxides as well, but at a lower rate than Fe^2+^.

In our study, Fe^2+^ chelating activity was determined using an adapted method of Gülçin et al. [13]. This method measures the absorbance of the Fe^2+^–ferrozine complex, which decreases in the presence of chelating agents, allowing the estimation of the metal chelating activity of CBD-enriched hemp oil, pure CBD, crude hemp oil and gallic acid. A concentration of 15 μg CBD/mL in CBD-enriched hemp oil exhibited 27.26 ± 0.2% chelating activity (Table 3), a value similar to previously published data for standard antioxidant compounds [13]. CBD-enriched hemp oil determines a decrease in absorbance similar to the control sample (methanol). Meanwhile, for the other samples we obtained very low values. Purified CBD and gallic acid had almost the same values, thus suggesting that the numerous compounds in hemp oil have synergistic antioxidant effects, higher than those of individual purified active substances.

**Figure 5:**
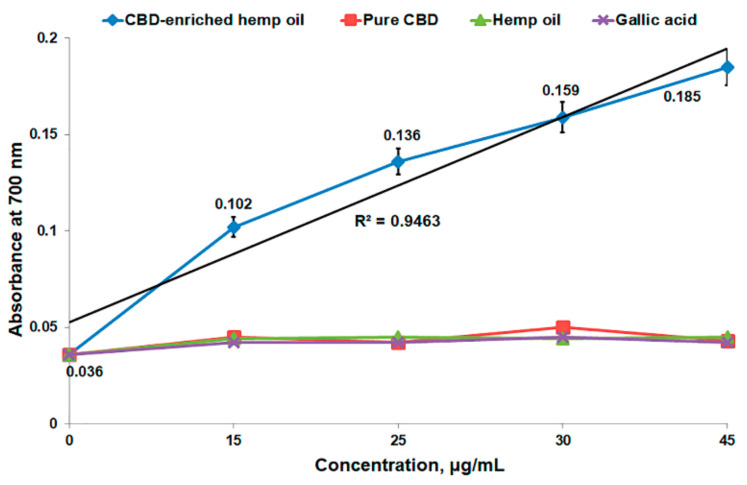
FRAP assay of CBD-enriched hemp oil (15–45 μg CBD/mL), pure CBD, crude hemp oil and gallic acid.

The antioxidant activity of CBD-enriched hemp oil, pure CBD, crude hemp oil and gallic acid of different concentrations was also determined through FRAP assay. FRAP assay is based on the direct reduction of ferricyanide to ferrocyanide by antioxidant compounds (i.e., phenolic derivatives), followed by the formation of Perl’s Prussian complex (intense blue color) in the presence of Fe^3+^ and the absorbance at 700 nm of the formed complex is directly proportional to the reducing capacity of the studied compounds [7]. In our case, CBD-enriched hemp oil in concentrations of 15–45 μg CBD/mL exhibited an effective reducing antioxidant power of 55% (Table 3), in a dose-dependent manner with r^2^ = 0.9463 (Figure 5), being in line with other phenolic derivatives [9,13]. The other studied compounds determined almost the same absorbance regardless of concentration, and the reducing antioxidant power was over 100% for pure CBD and almost 100% for crude hemp oil and gallic acid.

The antioxidant capacity of vegetal bioactive compounds can be estimated also by the compounds’ capacity to neutralize ROS with a short half-life, such as O_2_^●−^ (mildly reactive) and HO^●^ (highly reactive), which are generated in different metabolic processes and may lead to lipid peroxidation and tissue damage [13].

In our case, as it can be observed in Table 3, CBD-enriched hemp oil of 15 μg CBD/mL presented a high capacity toward the inactivation of O_2_^●−^ and HO^●^ super reactive radicals, agreeing with other published data [26,27,28], while for the other analyzed samples we obtained values around 1. Once again, it is confirmed that the isolated compounds have lower antioxidant activities compared to the parent hemp extract.

The last method applied for the determination of scavenging activity toward ROS was the lipid peroxidation assay, which is initiated by O_2_^●−^ and HO^●^ through free radical chain reactions [13]. The amount of lipid peroxides produced during the process can be measured using the ferric thiocyanate method. We determined a high lipid peroxidation inhibitory effect of CBD-enriched hemp oil of 15 μg CBD/mL over 4 days (59.77 ± 2%), 2.7 times higher than for crude hemp oil and 2.8 times higher than for gallic acid.

Taken together, these results show that CBD-enriched hemp oil has a high antioxidant activity, being capable of reducing iron, scavenging free radicals and inhibiting lipid peroxidation.

### 3.4. Cytotoxicity of CBD-Enriched Hemp Oil

Cytotoxicity on normal fibroblasts (NHDF) and malignant cell lines (MeWo, HeLa, HepG2 and HOS) of CBD-enriched hemp oil was determined using MTS assay after 48 h incubation. The CBD-enriched hemp oil samples of different CBD concentrations used for cell treatments were obtained by serial dilutions of oil extracted from *C. sativa* L., cultivated in north-eastern Romania, with maximum yield of CBDA decarboxylation.

**Figure 6:**
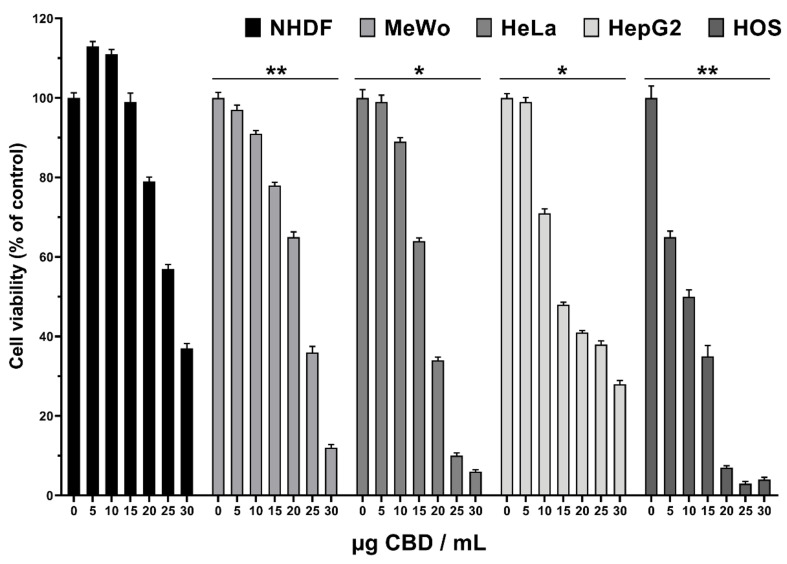
Cytotoxicity of CBD-enriched hemp oil on normal dermal fibroblasts (NHDF), malignant melanoma (MeWo), adenocarcinoma (HeLa), hepatocellular carcinoma (HepG2) and osteosarcoma (HOS) cells. Control cells were incubated with complete cell culture medium (represented as 0 µg CBD/mL). Treated cell viability was expressed as a percentage of control cells’ viability. Data were represented as means ± standard error of the mean. * *p* < 0.05, ** *p* < 0.01 vs. NHDF cells (one-way ANOVA: MeWo *p* = 0.0052, HeLa *p* = 0.0149, HepG2 *p* = 0.0400, HOS *p* = 0.0065).

The results show that CBD-enriched hemp oil promoted the proliferation of normal fibroblasts at concentrations up to 15 µg CBD/mL, followed by cytotoxic effects at higher concentrations (Figure 6), being in agreement with other published data [29,30,31,32].

Regarding the cell viability of malignant cells, its decrease started at a concentration of 5 µg CBD/mL, in a more pronounced or lesser manner, depending on the malignant cell line. Thus, the viability of the HOS cell line decreased dramatically even at a concentration of 5 µg CBD/mL, while for HepG2, HeLa and MeWo cells at 10 µg CBD/mL (Figure 6).

Taking into consideration these results, it can be concluded that the range of optimal concentrations is between 5 and 15 µg CBD/mL as fibroblast proliferation is not affected, while a decrease in viability of cancer cells can be observed.

Additionally, an important parameter in studying the anticancer effect of different compounds is the half-maximal inhibitory concentration on cell viability (IC_50_), meaning in our case the amount of CBD necessary to decrease the cell viability by 50%. Another important parameter is the selectivity index (SI) of compounds toward cell lines. When SI values are higher than 2, a good selectivity of the compound toward a certain cancer line can be considered and, also, the higher the value, the better selectivity to the cell line is considered, while SI values < 2 suggest general toxicity, thus affecting normal cells as well [17]. SI was calculated, as suggested by Badisa et al. [17], as being SI = IC_50_ normal cells/IC_50_ studied cancer cells.

Data presented in Table 4 suggest that CBD-enriched hemp oil is most effective on HOS cells (SI = 3.16), with the lowest IC_50_ value (8.42) being at least three times lower than the one obtained for NHDF cells (IC_50_ = 26.65), suggesting that CBD-enriched hemp oil is selective towards osteosarcoma and could be used as an alternative or adjuvant treatment. To the best of our knowledge, CBD’s or hemp oil’s cytotoxic effects have not been investigated in osteosarcoma. However, some research groups have determined that a synthetic cannabinoid (WIN 55,212-2) induced cell cycle arrest and endoplasmic reticulum stress [33] and had antiproliferative effects alone or combined with Adriamycin as a cytostatic drug on osteosarcoma cell lines [34].

CBD-enriched hemp oil presented similar IC_50_ and SI values in HeLa and HepG2 cells (Table 4). Interestingly, from the data presented above it can be observed that CBD-enriched hemp oil promoted proliferation of normal fibroblasts at concentrations up to 15 µg CBD/mL, which is close to the IC_50_ for HeLa and HepG2 cells and almost twice as high as the IC_50_ for HOS cells. Taking into consideration other published data, showing that *C. sativa* extracts and individual CBD exhibit anti-proliferative effects in HeLa and HepG2 cells [30,31], and the CBD pretreatment enhanced cisplatin cytotoxicity in HepG2 cells [35], we can recommend CBD-enriched hemp oil as a pretreatment or adjuvant in adenocarcinoma (HeLa) and hepatocellular carcinoma (HepG2) therapies. On account of the obtained results (Figure 6 and Table 4), malignant melanoma (MeWo) cells are the least sensitive to CBD-enriched hemp oil, but the obtained data recommend the use of CBD-enriched hemp oil in topical administration in melanoma treatment. This finding is also sustained by the results of Blázquez et al. [32], who reported that cannabinoids selectively inhibit melanoma cells’ growth, and of Simmerman et al. [36], who reported that CBD reduces tumor growth and size and improves survival time and quality of life in a mouse model of melanoma, similar to results obtained for cisplatin.

### 3.5. Evaluation of CBD-Enriched Hemp Oil-Induced Apoptosis

Evaluation of apoptosis was done by morphological analysis, AO/EB staining and apoptosis-related genes’ expression in normal fibroblasts and malignant cell lines treated with various concentrations of CBD-enriched hemp oil for 48 h.

#### 3.5.1. Morphological Analysis

**Figure 7:**
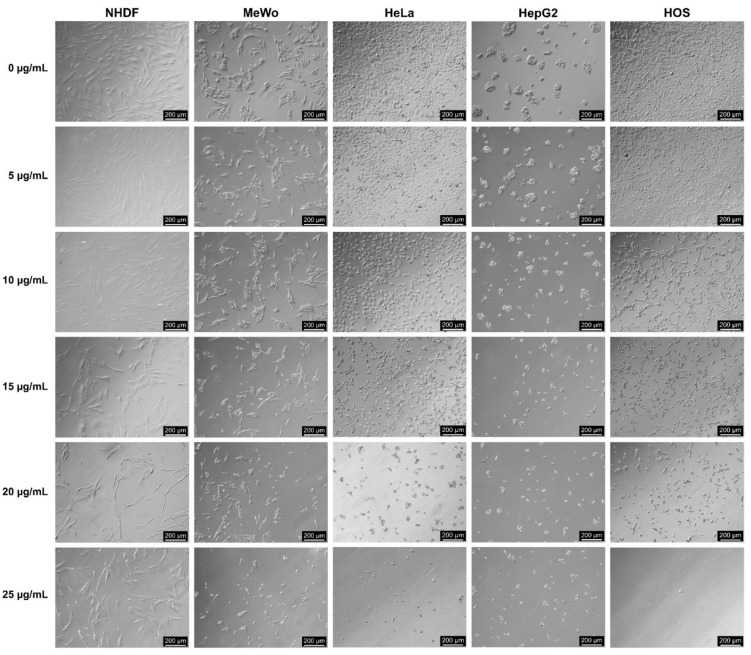
Morphological analysis of normal dermal fibroblasts (NHDF), malignant melanoma (MeWo), adenocarcinoma (HeLa), hepatocellular carcinoma (HepG2) and osteosarcoma (HOS) cells treated with CBD-enriched hemp oil at 5, 10, 15, 20 and 25 µg CBD/mL for 48 h. Control cells were incubated with complete cell culture medium (represented as 0 µg CBD/mL).

Some morphological changes and a slightly decreased number of NHDF cells were observed starting from 10 µg CBD/mL compared to control cells (0 µg CBD/mL), and, by increasing the CBD concentrations, cell shrinkage and detachment, cytoplasmic condensation and vesicle formation were observed (Figure 7). MeWo cells showed vesicle formation at 10 µg CBD/mL, aggravated by increasing CBD concentrations which promoted cell shrinkage and detachment (Figure 7), being in agreement with previously published data [29]. HeLa cells exhibited cytoplasmic condensation and vesicle formation starting from 10 µg CBD/mL, followed by accelerated dose-dependent cell rounding, shrinkage and detachment (Figure 7). Furthermore, almost complete cell detachment from the culture plate was observed for HeLa cells at 25 µg CBD/mL (Figure 7). HepG2 cells showed spheroid disaggregation and cell detachment starting from 10 µg/mL, amplified in a dose-dependent manner (Figure 7). HOS cells exhibited some cell rounding and a small decrease in cell number at 5 µg CBD/mL, followed by increased morphological changes at 10 µg CBD/mL, such as cytoplasmic condensation, vesicle formation, cell rounding and detachment (Figure 7). Furthermore, 15 µg CBD/mL induced extensive cell rounding and shrinkage in HOS cells, followed by vesicle formation at 20 µg CBD/mL and complete cell detachment at 25 µg CBD/mL (Figure 7).

#### 3.5.2. Acridine Orange/Ethidium Bromide (AO/EB) Staining for Apoptosis

AO/EB staining allows visualization of apoptotic body formation and nuclear changes characteristic of apoptosis [37]. Acridine orange stains both live and dead cells, while ethidium bromide stains only cells that have lost membrane integrity [37]. Therefore, live cells appear uniformly in green, while apoptotic cells exhibit chromatin condensation (bright green nuclei) and nuclear fragmentation, as well as orange-red staining of apoptotic bodies [37].

**Figure 8:**
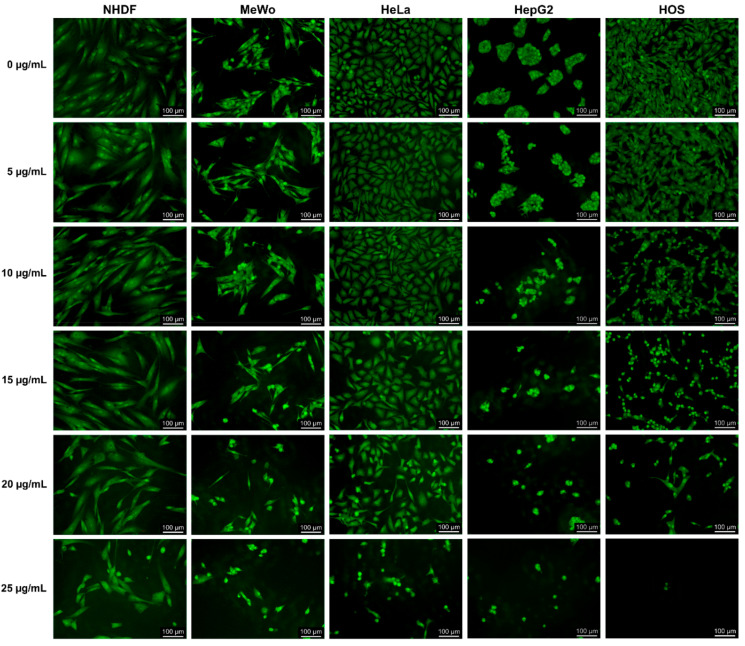
AO/EB staining of normal dermal fibroblasts (NHDF), malignant melanoma (MeWo), adenocarcinoma (HeLa), hepatocellular carcinoma (HepG2) and osteosarcoma (HOS) cells treated with CBD-enriched hemp oil at 5, 10, 15, 20 and 25 µg CBD/mL for 48 h. Control cells were incubated with complete cell culture medium (represented as 0 µg CBD/mL).

In order to determine the capacity of CBD-enriched hemp oil to induce apoptosis in different malignant cell lines, MeWo, HeLa, HepG2, HOS and NHDF cells were treated with different doses of CBD-enriched hemp oil for 48 h and AO/EB staining was applied (Figure 8), according to a method proposed by Ribble et al. [18]. No significant apoptosis was observed in the control cells (0 µg CBD/mL). We remark that the orange-red staining is more distinctive through the microscope than in the captured images.

From Figure 8 it can be observed that fibroblasts showed the first signs of apoptosis (such as chromatin condensation and vesicle formation) at concentrations of 20 µg CBD/mL, and more pronounced cytoplasmic and nuclear changes at 25 µg/mL were produced, while malignant cells had different behavior in a CBD dose-dependent manner.

MeWo cells (Figure 8) displayed few apoptotic bodies (stained orange-red), chromatin condensation and cytoplasmic shrinkage at 5 µg CBD/mL followed by nuclear fragmentation at 10 µg CBD/mL. The degree of apoptosis in MeWo cells increased, leading to the decreasing number of cells in a dose-dependent manner.

HeLa cells (Figure 8) exhibited cytoplasmic condensation starting from 10 µg CBD/mL, aggravated with the increasing CBD concentrations. Incubation of HeLa cells with 20 µg CBD/mL promoted nuclear fragmentation and vesicle formation, followed by increased cell death and detachment at 25 µg/mL, being in line with the results of Lukhele and Motadi [30] who demonstrated that CBD and different *C. sativa* extracts induce apoptosis in HeLa cells.

Starting at 5 µg/mL, chromatin condensation and nuclear fragmentation occurred in HepG2 cells (Figure 8), accompanied by spheroid disaggregation and cell detachment, also in a dose-dependent manner. Concentrations of 20 and 25 µg CBD/mL induced evident apoptotic features and almost complete cell detachment in HepG2 cells. It should be mentioned that Manosroi et al. [31] reported as well the induction of apoptosis in HeLa and HepG2 cells by hemp leaf extracts (without information on cannabinoid content).

HOS cells exhibited chromatin condensation, cell shrinkage and detachment starting from 10 µg CBD/mL, as well as apoptotic bodies’ formation (stained red) starting from 15 µg CBD/mL (Figure 8). Moreover, the incubation of HOS cells with 25 µg CBD/mL resulted in a complete cell detachment from culture plates. To the best of our knowledge, apoptosis induced by CBD or hemp oil has not been reported for osteosarcoma cells.

#### 3.5.3. Apoptosis-Related Genes’ Expression

The gene expressions of BAX, BCL2, TP53 and MDM2 were determined relative to 18S (as a reference gene) by real-time quantitative PCR (see Section 2.11 and Table 1), and calculated using the 2^−ΔΔCq^ method [20]. For this purpose, cells were incubated with fresh complete medium (control, 0 µg CBD/mL) or specific concentrations of CBD-enriched hemp oil (IC_50_ calculated for each cell line) for 48h (see Section 2.11).

**Figure 9:**
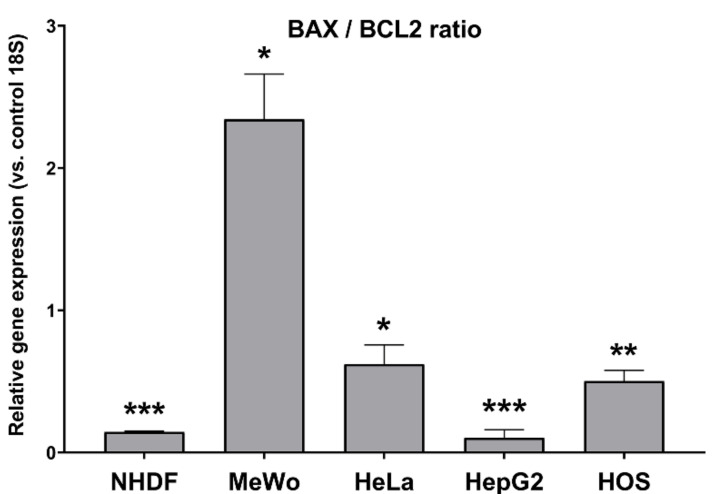
BAX/BCL2 gene expression ratio in normal dermal fibroblasts (NHDF), malignant melanoma (MeWo), adenocarcinoma (HeLa), hepatocellular carcinoma (HepG2) and osteosarcoma (HOS) cells treated with cell line-specific IC_50_ of CBD-enriched hemp oil for 48 h. Control cells were incubated with complete cell culture medium. Experiments were done in triplicate, obtained data were expressed as mean 2^−ΔΔCq^ values (vs. 18S of control cells) ± standard error of the mean. * *p* < 0.05, ** *p* < 0.01, *** *p* < 0.001 vs. control cells (NHDF *p* < 0.0001, MeWo *p* = 0.013391, HeLa *p* = 0.049447, HepG2 *p* = 0.000092, HOS *p* = 0.002741).

Besides gene expression measurements of BAX and BCL2 in cells treated with IC_50_ of CBD-enriched hemp oil for 48 h (see Section 2.11), the BAX/BCL2 ratios were calculated to determine the cells’ resistance to apoptosis. It is known that BAX promotes cell death, while BCL2 inhibits the activity of BAX and prevents apoptosis [38], therefore a BAX/BCL2 ratio < 1 indicates that the cells are resistant to apoptosis, while a ratio > 1 implies a sensitivity to apoptotic stimuli [39,40]. As it can be observed in Figure 9, when the cell lines were treated with IC_50_ doses of CBD-enriched hemp oil for 48 h, the BAX/BCL2 ratios corresponding to NHDF, HeLa, HepG2 and HOS lines were less than 1, and the BAX/BCL2 ratio was higher than 1 in the case of the MeWo cell line with *p* < 0.05. This suggests that HeLa, HepG2 and HOS cells are resistant to apoptosis, but they nonetheless undergo apoptosis at lower concentrations of CBD-enriched hemp oil compared to MeWo cells, as proven by morphological analysis and AO/EB staining. The results obtained for MeWo cells are in agreement with previously published data [39] and could have implications in cancer treatment, due to the increased susceptibility of CBD-pretreated melanoma cells to standard chemotherapy.

The tumor suppressor p53 plays a major role in regulating the cellular stress response, activating the transcription of different genes involved in cell cycle arrest and apoptosis [41]. p53 up-regulates the expression of the BAX gene and down-regulates the expression of the BCL2 gene, altering the BAX/BCL2 ratio and influencing the cell’s sensitivity to apoptotic stimuli [39]. Furthermore, p53 and the proto-oncogene MDM2 are linked through an autoregulatory feedback loop in which the former up-regulates MDM2 expression and the latter down-regulates p53, leading to an inhibition of p53-dependent apoptosis [41]. In cancer cells, p53’s gene is often mutated, or wild-type p53’s function is hindered by the overexpression of MDM2, leading to the inhibition of tumor-suppressive pathways and to resistance to apoptotic stimuli [41].

**Figure 10:**
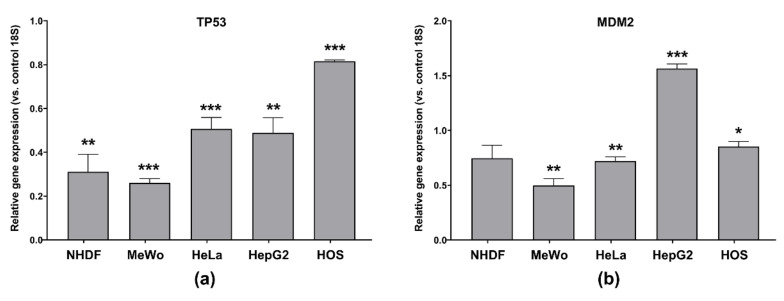
(**a**) TP53 gene expression; (**b**) MDM2 gene expression in normal dermal fibroblasts (NHDF), malignant melanoma (MeWo), adenocarcinoma (HeLa), hepatocellular carcinoma (HepG2) and osteosarcoma (HOS) cells treated with cell line-specific IC_50_ of CBD-enriched hemp oil for 48 h. Control cells were incubated with complete cell culture medium. Experiments were done in triplicate, obtained data were expressed as mean 2^−ΔΔCq^ values (vs. 18S of control cells) ± standard error of the mean. * *p* < 0.05, ** *p* < 0.01, *** *p* < 0.001 vs. control cells (TP53: NHDF *p* = 0.001014, MeWo *p* = 0.000003, HeLa *p* = 0.000669, HepG2 *p* = 0.001736, HOS *p* = 0.000011; MDM2: NHDF *p* = 0.065319, MeWo *p* = 0.001181, HeLa *p* = 0.002125, HepG2 *p* = 0.000358, HOS *p* = 0.033260).

In our case, gene expression of TP53 and MDM2 in cells treated with IC_50_ of CBD-enriched hemp oil for 48 h revealed that CBD-enriched hemp oil induced significant down-regulation of TP53 in all cell lines (Figure 10a). This suggests that CBD-enriched hemp oil promoted apoptosis in the studied malignant cell lines through p53-independent mechanisms that are in contradiction with the results of Lukhele and Motadi [30], who report that CBD and different *C. sativa* extracts up-regulate p53 protein expression in HeLa cells. Moreover, our results show that the administration of CBD-enriched hemp oil at IC_50_ concentrations induced significant down-regulation of MDM2 gene expression in MeWo and HeLa cells, but significant up-regulation of MDM2 was noted in HepG2 cells (Figure 10b). In agreement with our results in HepG2 cells, Alharris et al. [42] reported that CBD down-regulates the TP53 gene and protein expression and up-regulates MDM2 expression in neuroblastoma cells. Since overexpression of MDM2 has been linked to metastasis, resistance to chemotherapy and poor prognosis, a possible avenue in cancer treatment could be the inhibition of MDM2 expression or function [41]. Due to antagonist results of CBD and hemp oil effects on TP53 and MDM2 gene expression, more data are needed to elucidate this mechanism in other cancer types.

### 3.6. Assessment of Oxidative Stress Induced by CBD-Enriched Hemp Oil

It has been shown that CBD induces apoptosis by stimulating ROS production and, consequently, increasing the antioxidant enzymes’ activity [6]. In order to determine the induction of oxidative stress in CBD-enriched hemp oil-treated cells, the intracellular H_2_O_2_ production and gene expression of some key antioxidant enzymes were measured.

#### 3.6.1. Intracellular H_2_O_2_ Production

**Figure 11:**
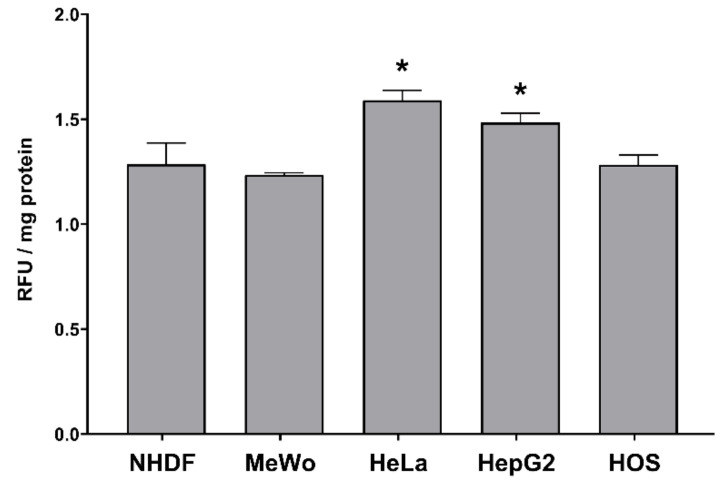
Intracellular H_2_O_2_ production in normal dermal fibroblasts (NHDF), malignant melanoma (MeWo), adenocarcinoma (HeLa), hepatocellular carcinoma (HepG2) and osteosarcoma (HOS) cells treated with cell line-specific IC_50_ doses of CBD-enriched hemp oil for 24 h. Control cells were incubated with complete cell culture medium. Experiments were done in triplicate, obtained data were expressed as relative fluorescence units (RFU)/mg protein, expressed as fold change of control values and represented as means ± standard error of the mean. * *p* < 0.05 vs. control cells (NHDF *p* = 0.058216, MeWo *p* = 0.160015, HeLa *p* = 0.011617, HepG2 *p* = 0.020345, HOS *p* = 0.055959).

The H_2_O_2_ production was determined in different malignant cell lines (MeWo, HeLa, HepG2 and HOS) and normal fibroblasts (NHDF) treated with cell line-specific IC_50_ of CBD-enriched hemp oil (Table 4) for 24 h. Interestingly, CBD-enriched hemp oil induced an increased production of H_2_O_2_ in all cell lines compared to untreated (control) cells, and it was significantly higher in HeLa and HepG2 cells (Figure 11). This result is in direct opposition to the cell-free antioxidant activity results that we obtained (see Section 3.3). Although CBD is known to exert antioxidative effects in different inflammatory conditions by modulating antioxidant enzymes’ activity and ROS production [9], it can induce ROS production in cancer cells as a means to induce apoptosis [6]. Our results show also that CBD-enriched hemp oil induces a slight increase in H_2_O_2_ production in NHDF cells at IC_50_ doses, in contrast to the findings of Massi et al. [43] who report that CBD induces time-dependent ROS production in glioma cells, but not in normal glial cells. In conclusion to this section, we can deduce that CBD can act as a producer of H_2_O_2_ in cancer cells, but more extensive studies need to be undertaken.

#### 3.6.2. Antioxidant Enzymes’ Gene Expression

SOD1 catalyzes the conversion of the highly reactive O_2_^●−^ to the less reactive H_2_O_2_, which can be further transformed by CAT into water and molecular oxygen or reduced by GPX1 using glutathione, while GSR restores intracellular glutathione [44]. In cultured cells, the addition of H_2_O_2_ causes a dose-dependent increase in SOD1, CAT and GPX1 gene expression [44].

**Figure 12:**
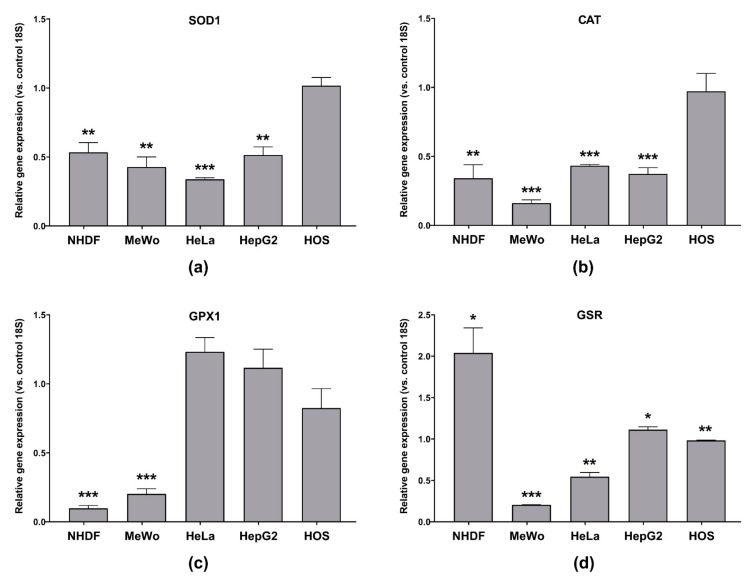
Gene expression of: (**a**) SOD1; (**b**) CAT; (**c**) GPX1; (**d**) GSR in normal dermal fibroblasts (NHDF), malignant melanoma (MeWo), adenocarcinoma (HeLa), hepatocellular carcinoma (HepG2) and osteosarcoma (HOS) cells treated with cell line-specific IC_50_ of CBD-enriched hemp oil for 48 h. Control cells were incubated with complete cell culture medium. Experiments were done in triplicate, obtained data were expressed as mean 2^−ΔΔCq^ values (vs. 18S of control cells) ± standard error of the mean. * *p* < 0.05, ** *p* < 0.01, *** *p* < 0.001 vs. control cells (SOD1: NHDF *p* = 0.003067, MeWo *p* = 0.001484, HeLa *p* < 0.000001, HepG2 *p* = 0.001186, HOS *p* = 0.796912; CAT: NHDF *p* = 0.002620, MeWo *p* = 0.000004, HeLa *p* < 0.000001, HepG2 *p* = 0.000146, HOS *p* = 0.821580; GPX1: NHDF *p* = 0.000002, MeWo *p* = 0.000027, HeLa *p* = 0.057635, HepG2 *p* = 0.337276, HOS *p* = 0.267738; GSR: NHDF *p* = 0.019436, MeWo *p* < 0.000001, HeLa *p* = 0.001320, HepG2 *p* = 0.026340, HOS *p* = 0.006656).

To the best of our knowledge, the effect of CBD-enriched hemp oil on antioxidant enzymes’ gene expression in MeWo, HeLa, HepG2 and HOS cells has not been investigated and, as a result, in this study, gene expression of SOD1, CAT, GPX1 and GSR in cells treated with IC_50_ doses of CBD-enriched hemp oil, specific to each cell line (Table 4) for 48 h, was measured (Figure 12). From Figure 12a it can be observed that SOD1 gene expression was decreased in NHDF, MeWo, HeLa and HepG2 cells treated with CBD-enriched hemp oil of specific IC_50_ concentrations, suggesting that H_2_O_2_ is produced mainly by other SODs. Moreover, Usami et al. [45] reported that in mouse hepatic 105,000× *g* supernatants, CBD decreased SOD activity. Additionally, in NHDF and MeWo cells treated with CBD-enriched hemp oil, CAT and GPX1 gene expression (Figure 12b,c) were significantly reduced, suggesting that H_2_O_2_ is inactivated by different mechanisms. Meanwhile, in treated HeLa and HepG2 cells (Figure 12b,c), CAT gene expression was also significantly decreased, but GPX1 expression was slightly increased, suggesting that GPX1, rather than CAT, inactivates H_2_O_2_ produced after CBD-enriched hemp oil treatment, being in correlation with significantly increased levels of H_2_O_2_ in treated cells. Similar results were reported by Usami et al. [45], by the fact that they showed that in mouse hepatic 105,000× *g* supernatants, CBD decreased CAT activity and increased GPX1 and GSR activities.

In normal cells (NHDF), after CBD-enriched hemp oil treatment for 48 h, GPX1 gene expression was significantly reduced (Figure 12c), but GSR gene expression was increased (Figure 12d). This finding suggests a greater cellular resilience to oxidative processes through an increase in glutathione levels. In the meantime, MeWo cells treated with CBD-enriched hemp oil presented significantly decreased GPX1 and GSR gene expression, suggesting their susceptibility to oxidative stress. This finding, together with MeWo cells’ sensitivity to apoptotic stimuli, suggests that CBD-enriched hemp oil induces apoptosis in MeWo cells by ROS-related mechanisms.

In treated HOS cells, SOD1, CAT, GPX1 and GSR gene expressions were largely unaffected by CBD-enriched hemp oil treatment (Figure 12). If we combine these results with the fact that a small increase in H_2_O_2_ production was observed, an important conclusion can be drawn, namely that CBD-enriched hemp oil induces apoptosis in HOS cells by ROS-independent mechanisms.

## 4. Conclusions

In conclusion, hemp oil was extracted with ethanol at room temperature from *C. sativa* L. denoted KC Dora, cultivated in north-eastern Romania, and after vacuum evaporation of the solvent, a yield of 5.09% from vegetal material was obtained. The content and the purity of CBD in extracted hemp oil were determined by flash chromatography and ^1^H- and ^13^C-NMR, respectively. We found the most efficient decarboxylation conditions for CBDA from hemp oil (90 °C, for 1 or 2 h), which yields hemp oil enriched in CBD without significant degradation of bioactive compounds.

The obtained CBD-enriched hemp oil was capable of reducing iron, scavenging free radicals and inhibiting lipid peroxidation in oxidative conditions, suggesting that it could protect against oxidative damage in normal cells. Conversely, CBD-enriched hemp oil induced ROS production in cancer cells, but more extensive studies need to be undertaken in order to elucidate the exact mechanisms. Furthermore, CBD-enriched hemp oil promoted the proliferation of normal fibroblasts when the concentration of CBD was up to 15 µg/mL, while being cytotoxic for cancer cells. To the best of our knowledge, this is the first study to investigate the cytotoxic effects of CBD or hemp oil on osteosarcoma cells, and the results show that CBD-enriched hemp oil was selective for osteosarcoma cells and induced apoptosis in a dose-dependent manner, by p53- and ROS-independent mechanisms.

CBD-enriched hemp oil promoted apoptosis in the studied malignant cell lines through p53-independent mechanisms, in contradiction with other published data [30]. Due to antagonistic results of CBD and hemp oil effects on TP53 and MDM2 gene expression, more data are needed to elucidate this mechanism in different cancer types.

To the best of our knowledge, the effect of CBD-enriched hemp oil on antioxidant enzymes’ gene expression in MeWo, HeLa, HepG2 and HOS cells has not been investigated. Our study revealed that CBD-enriched hemp oil modulated the gene expression of antioxidant enzymes in a cell line-specific manner. Our findings suggest normal fibroblasts treated with CBD-enriched hemp oil have greater cellular resilience to oxidative stress, mainly through an increase in glutathione levels, in contrast with malignant melanoma cells. Moreover, our results suggest that CBD-enriched hemp oil induces apoptosis in MeWo cells by ROS-related mechanisms. Additionally, our results suggest that CBD-enriched hemp oil could be used as a pretreatment or adjuvant in adenocarcinoma (HeLa) and hepatocellular carcinoma (HepG2) therapies, as well as in topical administration in melanoma treatment.

Our study aimed to extend the knowledge in the field, and our results give new insights on the biological effects of CBD-enriched hemp oil, recommending it as adjuvant or treatment in various cancers, especially for osteosarcoma, with numerous health benefits and very few side effects. CBD-enriched hemp oil should be considered a versatile nutraceutical with applications in cancer management and treatment and, although CBD is the major pharmacologically active compound in CBD-enriched hemp oil, this product should be viewed as a complex mixture of bioactive molecules with synergic properties.

## Figures and Tables

**Table 1:** Primer sequences and amplicon size used for gene expression analysis by real-time PCR.

Gene	Gene ID	Sequence 5′-3′	Amplicon Size (bp)
18S	NR_145820.1	FW 5′ GGAGCCTGCGGCTTAATTTG RV 5′ CCACCCACGGAATCGAGAAA	100
BAX	NM_138761.3	FW 5′ TCATGGGCTGGACATTGGAC RV 5′ GCGTCCCAAAGTAGGAGAGG	96
BCL2	NM_000633.2	FW 5′ GCGGCCTCTGTTTGATTTCTC RV 5′ CTTGTGGCCCAGATAGGCA	100
TP53	NM_001276696.2	FW 5′ CAGCACATGACGGAGGTTGT RV 5′ TCATCCAAATACTCCACACGC	125
MDM2	NM_002392.5	FW 5′ CAGTAGCAGTGAATCTACAGGGA RV 5′ CTGATCCAACCAATCACCTGAAT	85
SOD1	NM_000454.4	FW 5′ GGAAGTCGTTTGGCTTGTGG RV 5′ GGGCCTCAGACTACATCCAAG	70
CAT	NM_001752.4	FW 5′ CTGACTACGGGAGCCACATC RV 5′ AGATCCGGACTGCACAAAGG	92
GPX1	NM_000581.4	FW 5′ CAGTCGGTGTATGCCTTCTCG RV 5′ GAGGGACGCCACATTCTCG	105
GSR	NM_000637.5	FW 5′ GCACTTGCGTGAATGTTGGA RV 5′ TCACATAGGCATCCCGCTTT	156

**Table 2:** Experimental results after decarboxylation in soft conditions of CBDA from hemp oil.

Nr. Crt.	Decarboxylation Conditions ^1^	CBD in 50 mg Hemp Oil (mg)	CBDA in 50 mg Hemp Oil (mg)	CBD + CBDA in 50 mg Hemp Oil (mg)	CBDin Hemp Oil (%) ^2^	CBDA in Hemp Oil (%) ^2^	CBD Transformation from CBDA (%) ^2^	CBDA + CBD Yield after Decarboxylation Process (%) ^2^	CBDA + CBD Degradation Yield (%) ^2^
	Crude oil	4.57	6.77	11.345	9.14	13.54	-	-	-
1	70 °C, 1 h	5.47	1.95	7.43	10.94	3.9	71.19	65.4	34.6
2	70 °C, 2 h	5.7	1.89	7.59	11.4	3.78	72.08	66.9	33.1
3	70 °C, 3 h	6.03	2.1	8.13	12.06	4.2	68.98	71.66	28.34
4	70 °C, 4 h	6.17	2.05	8.22	12.34	4.1	69.72	72.45	27.55
5	80 °C, 1 h	7.47	2.38	9.85	14.94	4.76	64.84	86.82	13.18
6	80 °C, 2 h	7.65	2.16	9.81	15.3	4.32	68.09	86.47	13.57
7	80 °C, 3 h	7.69	1.81	9.5	15.38	3.62	73.26	83.73	16.27
8	80 °C, 4 h	7.83	1.7	9.53	15.66	3.4	74.89	84.0	16.0
9	90 °C, 1 h	8.6	2.26	10.86	17.2	4.52	66.62	95.72	4.28
10	90 °C, 2 h	8.91	1.8	10.81	17.82	3.8	73.41	95.28	4.72
11	100 °C, 1 h	7.9	1.35	9.25	15.8	2.7	80.06	81.53	18.47

^1^ All reactions were performed at 500 mbar, 60 rpm. ^2^ The values are calculated in relation to the values obtained from crude oil.

**Table 3:** Antioxidant activity of CBD-enriched hemp oil, pure CBD, crude hemp oil and gallic acid.

Samples/Method	Fe^2+^ Chelating Activity (%)	FRAP Assay (%) ^1^	O_2_^●−^ Scavenging Activity (%)	HO^●^ Scavenging Ability (%)	Lipid Peroxidation Inhibitory Assay (%) ^2^
CBD-enriched hemp oil (15 μg CBD/mL)	27.26 ± 0.2	55.0 ± 1.3	69.1 ± 3	221.5 ± 2.56	59.77 ± 2.0
Pure CBD (15 μg/mL)	3.7 ± 0.1	104.6 ± 2.1	0.41 ± 0.01	167 ± 2.15	1.66 ± 0.04
Crude hemp oil (15 μg CBD/mL)	8.0 ± 0.03	97.7 ± 1.7	1.24 ± 0.03	20.69 ± 0.63	22.36 ± 1.18
Gallic acid (15 μg/mL)	3.8 ± 0.07	95.45 ± 1.3	1.23 ± 0.01	391.38 ± 5.67	21.33 ± 1.03

^1^ Compounds in concentrations of 15–45 μg CBD/mL. ^2^ over 4 days.

**Table 4:** IC_50_ and SI values of CBD-enriched hemp oil toward NHDF, MeWo, HeLa, HepG2 and HOS cell lines.

	NHDF	MeWo	HeLa	HepG2	HOS
IC_50_ (µg CBD/mL)	26.65	21.59	16.89	16.85	**8.42**
SI	-	1.23	1.58	1.58	**3.16**

## Data Availability

The data presented in this study are available on request from the corresponding author.

## References

[1] Fiorini D., Molle A., Nabissi M., Santini G., Benelli G., Maggi F. (2019). Valorizing industrial hemp (Cannabis sativa L.) by-products: Cannabidiol enrichment in the inflorescence essential oil optimizing sample pre-treatment prior to distillation. Ind. Crops Prod..

[2] Di Giacomo V., Recinella L., Chiavaroli A., Orlando G., Cataldi A., Rapino M., Di Valerio V., Politi M., Antolini M.D., Acquaviva A. (2021). Metabolomic profile and antioxidant/anti-inflammatory effects of industrial hemp water extract in fibroblasts, keratinocytes and isolated mouse skin specimens. Antioxidants.

[3] Orlando G., Adorisio S., Delfino D., Chiavaroli A., Brunetti L., Recinella L., Leone S., D’antonio M., Zengin G., Acquaviva A. (2021). Comparative investigation of composition, antifungal, and anti-inflammatory effects of the essential oil from three industrial hemp varieties from Italian cultivation. Antibiotics.

[4] Nagy D.U., Cianfaglione K., Maggi F., Sut S., Dall’Acqua S. (2019). Chemical Characterization of Leaves, Male and Female Flowers from Spontaneous Cannabis (Cannabis sativa L.) Growing in Hungary. Chem. Biodivers..

[5] Tomko A.M., Whynot E.G., Ellis L.D., Dupré D.J. (2020). Anti-cancer potential of cannabinoids, terpenes, and flavonoids present in cannabis. Cancers.

[6] Śledziński P., Zeyland J., Słomski R., Nowak A. (2018). The current state and future perspectives of cannabinoids in cancer biology. Cancer Med..

[7] Gülçin I. (2015). Fe3+–Fe2+ transformation method: An important antioxidant assay. Methods Mol. Biol..

[8] Lennicke C., Rahn J., Lichtenfels R., Wessjohann L.A., Seliger B. (2015). Hydrogen peroxide—Production, fate and role in redox signaling of tumor cells. Cell Commun. Signal..

[9] Atalay S., Jarocka-karpowicz I., Skrzydlewska E., Skrzydlewskas E. (2020). Antioxidative and anti-inflammatory properties of cannabidiol. Antioxidants.

[10] Citti C., Pacchetti B., Vandelli M.A., Forni F., Cannazza G. (2018). Analysis of cannabinoids in commercial hemp seed oil and decarboxylation kinetics studies of cannabidiolic acid (CBDA). J. Pharm. Biomed. Anal..

[11] Ocque A.J., Hagler C.E., DiFrancesco R., Lombardo J., Morse G.D. (2019). Development and validation of an assay to measure cannabidiol and Δ9-tetrahydrocannabinol in human EDTA plasma by UHPLC-MS/MS. J. Chromatogr. B Anal. Technol. Biomed. Life Sci..

[12] Escrivá Ú., Andrés-Costa M.J., Andreu V., Picó Y. (2017). Analysis of cannabinoids by liquid chromatography–mass spectrometry in milk, liver and hemp seed to ensure food safety. Food Chem..

[13] Gülçin I., Elmastaş M., Aboul-Enein H.Y. (2012). Antioxidant activity of clove oil—A powerful antioxidant source. Arab. J. Chem..

[14] Li X., Wang X., Chen D., Chen S. (2011). Antioxidant activity and mechanism of protocatechuic acid in Vitro. Funct. Foods Heal. Dis..

[15] Xiong L., Ni X., Niu L., Zhou Y., Wang Q., Khalique A., Liu Q., Zeng Y., Shu G., Pan K. (2019). Isolation and Preliminary Screening of a Weissella confusa Strain from Giant Panda (Ailuropoda melanoleuca). Probiotics Antimicrob. Proteins.

[16] Ahmed D., Khan M.M., Saeed R. (2015). Comparative analysis of phenolics, flavonoids, and antioxidant and antibacterial potential of methanolic, hexanic and aqueous extracts from Adiantum caudatum leaves. Antioxidants.

[17] Badisa R.B., Darling-Reed S.F., Joseph P., Cooperwood J.S., Latinwo L.M., Goodman C.B. (2009). Selective cytotoxic activities of two novel synthetic drugs on human breast carcinoma MCF-7 cells. Anticancer Res..

[18] Ribble D., Goldstein N.B., Norris D.A., Shellman Y.G. (2005). A simple technique for quantifying apoptosis in 96-well plates. BMC Biotechnol..

[19] Toma L., Sanda G.M., Deleanu M., Stancu C.S., Sima A.V. (2016). Glycated LDL increase VCAM-1 expression and secretion in endothelial cells and promote monocyte adhesion through mechanisms involving endoplasmic reticulum stress. Mol. Cell. Biochem..

[20] Schmittgen T.D., Livak K.J. (2008). Analyzing real-time PCR data by the comparative CT method. Nat. Protoc..

[21] Fathordoobady F., Singh A., Kitts D.D., Pratap Singh A. (2019). Hemp (Cannabis Sativa L.) Extract: Anti-Microbial Properties, Methods of Extraction, and Potential Oral Delivery. Food Rev. Int..

[22] Choi Y.H., Hazekamp A., Peltenburg-Looman A.M.G., Frédérich M., Erkelens C., Lefeber A.W.M., Verpoorte R. (2004). NMR assignments of the major cannabinoids and cannabiflavonoids isolated from flowers of Cannabis sativa. Phytochem. Anal..

[23] Petrović M., Debeljak Ž., Kezić N., Džidara P. (2015). Relationship between cannabinoids content and composition of fatty acids in hempseed oils. Food Chem..

[24] Borges R.S., Da Silva A.B.F. (2017). Cannabidiol as an Antioxidant. Handbook of Cannabis and Related Pathologies: Biology, Pharmacology, Diagnosis, and Treatment.

[25] Suktham T., Jones A., Soliven A., Dennis G.R., Shalliker R.A. (2019). A comparison of the performance of the cupric reducing antioxidant potential assay and the ferric reducing antioxidant power assay for the analysis of antioxidants using reaction flow chromatography. Microchem. J..

[26] Hacke A.C.M., Lima D., De Costa F., Deshmukh K., Li N., Chow A.M., Marques J.A., Pereira R.P., Kerman K. (2019). Probing the antioxidant activity of Δ9-tetrahydrocannabinol and cannabidiol in Cannabis sativa extracts. Analyst.

[27] Moccia S., Siano F., Russo G.L., Volpe M.G., La Cara F., Pacifico S., Piccolella S., Picariello G. (2020). Antiproliferative and antioxidant effect of polar hemp extracts (Cannabis sativa L., Fedora cv.) in human colorectal cell lines. Int. J. Food Sci. Nutr..

[28] Moldzio R., Pacher T., Krewenka C., Kranner B., Novak J., Duvigneau J.C., Rausch W.D. (2012). Effects of cannabinoids Δ(9)-tetrahydrocannabinol, Δ(9)-tetrahydrocannabinolic acid and cannabidiol in MPP+ affected murine mesencephalic cultures. Phytomedicine.

[29] Choi W.H., Park H.D., Baek S.H., Chu J.P., Kang M.H., Mi Y.J. (2008). Cannabidiol induces cytotoxicity and cell death via apoptotic pathway in cancer cell lines. Biomol. Ther..

[30] Lukhele S.T., Motadi L.R. (2016). Cannabidiol rather than Cannabis sativa extracts inhibit cell growth and induce apoptosis in cervical cancer cells. BMC Complement. Altern. Med..

[31] Manosroi A., Chankhampan C., Kietthanakorn B.O., Ruksiriwanich W., Chaikul P., Boonpisuttinant K., Sainakham M., Manosroi W., Tangjai T., Manosroi J. (2019). Pharmaceutical and cosmeceutical biological activities of hemp (cannabis sativa L var. sativa) leaf and seed extracts. Chiang Mai J. Sci..

[32] Blázquez C., Carracedo A., Barrado L., José Real P., Luis Fernández-Luna J., Velasco G., Malumbres M., Guzmán M., Blázquez C., Carracedo A. (2006). Cannabinoid receptors as novel targets for the treatment of melanoma. FASEB J..

[33] Notaro A., Sabella S., Pellerito O., Di Fiore R., De Blasio A., Vento R., Calvaruso G., Giuliano M. (2014). Involvement of PAR-4 in cannabinoid-dependent sensitization of osteosarcoma cells to TRAIL-induced Apoptosis. Int. J. Biol. Sci..

[34] Niu F., Zhao S., Xu C.Y., Sha H., Bi G.B., Chen L., Ye L., Gong P., Nie T.H. (2015). Potentiation of the antitumor activity of adriamycin against osteosarcoma by cannabinoid WIN-55,212-2. Oncol. Lett..

[35] Kosgodage U.S., Mould R., Henley A.B., Nunn A.V., Guy G.W., Thomas E.L., Inal J.M., Bell J.D., Lange S. (2018). Cannabidiol (CBD) is a novel inhibitor for exosome and microvesicle (EMV) release in cancer. Front. Pharmacol..

[36] Simmerman E., Qin X., Yu J.C., Baban B. (2019). Cannabinoids as a Potential New and Novel Treatment for Melanoma: A Pilot Study in a Murine Model. J. Surg. Res..

[37] Kasibhatla S. (2006). Acridine Orange/Ethidium Bromide (AO/EB) Staining to Detect Apoptosis. Cold Spring Harb. Protoc..

[38] Kulsoom B., Shamsi T.S., Afsar N.A., Memon Z., Ahmed N., Hasnain S.N. (2018). Bax, Bcl-2, and Bax/Bcl-2 as prognostic markers in acute myeloid leukemia: Are we ready for bcl-2-directed therapy?. Cancer Manag. Res..

[39] Raisova M., Hossini A.M., Eberle J., Riebeling C., Wieder T., Sturm I., Daniel P.T., Orfanos C.E., Geilen C.C. (2001). The Bax/Bcl-2 ratio determines the susceptibility of human melanoma cells to CD95/Fas-mediated apoptosis. J. Investig. Dermatol..

[40] Khodapasand E., Jafarzadeh N., Farrokhi F., Kamalidehghan B., Houshmand M. (2015). Is Bax/Bcl-2 ratio considered as a prognostic marker with age and tumor location in colorectal cancer?. Iran. Biomed. J..

[41] Nag S., Qin J., Srivenugopal K.S., Wang M., Zhang R. (2013). The MDM2-p53 pathway revisited. J. Biomed. Res..

[42] Alharris E., Singh N.P., Nagarkatti P.S., Nagarkatti M. (2019). Role of miRNA in the regulation of cannabidiol-mediated apoptosis in neuroblastoma cells. Oncotarget.

[43] Massi P., Vaccani A., Bianchessi S., Costa B., Macchi P., Parolaro D. (2006). The non-psychoactive cannabidiol triggers caspase activation and oxidative stress in human glioma cells. Cell. Mol. Life Sci..

[44] Matés J.M., Pérez-Gómez C., De Castro I.N. (1999). Antioxidant enzymes and human diseases. Clin. Biochem..

[45] Usami N., Yamamoto I., Watanabe K. (2008). Generation of reactive oxygen species during mouse hepatic microsomal metabolism of cannabidiol and cannabidiol hydroxy-quinone. Life Sci..

